# BMDM-derived ORP8 suppresses lipotoxicity and inflammation by relieving endoplasmic reticulum stress in mice with MASH

**DOI:** 10.1186/s10020-025-01275-6

**Published:** 2025-05-30

**Authors:** Yi Chen, Kangjie Xie, Caiyang Chen, Xihui Wang, Chenchen Ma, Zhangxiang Huang, Yingfu Jiao, Weifeng Yu

**Affiliations:** 1 Department of Anesthesiology, Renji Hospital, Jiaotong University School of Medicine, No. 160, Pujian Road, Pudong New District, Shanghai, 200217 China; 2 Key Laboratory of Anesthesiology, Shanghai Jiao Tong University, Ministry of Education, Shanghai, 200217 China; 3 Department of Anesthesiology, The First Affiliated Hospital of Wenzhou Medical University, Wenzhou, Zhejiang 310022 China; 4 Department of Anesthesiology, Hangzhou Institute of Medicine (HIM), Zhejiang Cancer Hospital, Chinese Academy of Sciences, Hangzhou, Zhejiang 310022 China; 5 Department of Pain Management, The First Affiliated Hospital of Kunming Medical University, Kunming, Yunnan 650032 China

**Keywords:** Bone marrow-derived macrophages, Endoplasmic reticulum stress, Extracellular vesicles, Lipotoxicity, Metabolic dysfunction-associated steatohepatitis

## Abstract

**Background and aims:**

Metabolic dysfunction-associated steatohepatitis (MASH) is one of the most common chronic liver diseases worldwide, and specific treatment modalities are lacking. Accumulating evidence suggests that hepatic inflammation plays a key role in the progression from hepatic steatosis to MASH. Macrophages, especially anti-inflammatory macrophages, serve as natural immune cells that maintain homeostasis in the immune microenvironment. Here, we aimed to reveal the role of anti-inflammatory macrophages in MASH and investigate the underlying mechanism involved.

**Methods & results:**

Extracellular vesicles (EVs) were isolated from the supernatant of anti-inflammatory bone marrow-derived macrophages (BMDMs) by ultracentrifugation, and their protein profile was characterized by liquid chromatography–tandem mass spectrometry (LC‒MS/MS) analysis. Murine hepatocytes were stimulated with palmitic acid (PA) followed by treatment with EVs or oxysterol-binding protein-related protein 8 (ORP8/Osbpl8) shRNA. C57BL/6 mice were fed a methionine- and choline-deficient (MCD) diet for 3 weeks to establish MASH. The mice were then treated with EVs or shRNA-encoding AAV. In vitro and ex vivo experiments revealed that extracellular vesicles derived from anti-inflammatory BMDMs inhibited inflammatory responses and alleviated lipotoxicity during MASH. We identified Osbpl8 as a vital component of M2-BMDMs by LC–MS/MS analysis and found that Osbpl8 remodels lipid metabolism by inhibiting excessive IRE1α-XBP1-related ER stress. Furthermore, Osbpl8-enriched M2-BMDM-EVs promoted anti-inflammatory and antilipotoxic effects and could be a novel therapeutic target for the clinical treatment of MASH.

**Conclusions:**

Our findings indicate that Osbpl8 derived from EVs secreted by anti-inflammatory BMDMs plays important roles in intercellular communication between macrophages and hepatocytes, revealing a novel regulatory mechanism of macrophage homoeostasis in MASH.

**Supplementary Information:**

The online version contains supplementary material available at 10.1186/s10020-025-01275-6.

## Introduction

In recent years, due to dietary habits and lifestyle changes, metabolic dysfunction-associated steatotic liver disease (MASLD, formerly known as non-alcoholic fatty liver disease [NAFLD]) has become a common chronic liver disease worldwide (Younossi 2019; Younossi et al. 2019; Rinella et al. 2023). MASLD can progress from simple steatosis to more severe metabolic dysfunction-associated steatohepatitis (MASH, formerly known as non-alcoholic steatohepatitis [NASH]), which is characterized by the abnormal accumulation of fat in liver tissue. Excessive fatty acids promote de novo synthesis of lipids and beta-oxidation of free fatty acids, which leads to oxidative stress and hepatic inflammation (Sheka et al. 2020; Loomba et al. 2021). Furthermore, obesity-related MASH is currently the third leading cause of liver transplantation and is expected to become the principal cause of liver transplantation in the developed world (Samji et al. 2019). Rezdiffra is the only Food and Drug Administration (FDA) approved therapy and works as an activator of a thyroid hormone receptor-beta which partially decreases steatosis (Kokkorakis et al. 2024; Keam and Resmetirom 2024). As such,, the discovery of effective intervention targets for MASH is urgently needed.

Recently, factors involved in the relationship among the gut microbiota, metabolic syndrome and immune-mediated disorders have been suggested to also be involved in the development and progression of MASH (Vuppalanchi et al. 2021; Bessone et al. 2019). Lipotoxicity and oxidative stress are considered central to the transition to MASH and fibrosis. Reactive oxygen species-mediated hepatic fibrosis is caused by the excessive production of ROS in the liver, which leads to peroxidative damage in hepatocytes (Peiseler et al. 2022). If not treated promptly, liver cirrhosis can develop and ultimately lead to hepatocellular carcinoma.

Key features of MASH include the activation of resident immune cells, particularly Kupffer cells, and the recruitment of macrophages, B cells, natural killer (NK) cells, and CD4^+^ and CD8^+^ T cells, which contribute to inflammation and the persistent cycle of tissue damage and repair (Peiseler et al. 2022; Schuster et al. 2018). Macrophages serve as natural immune cells that maintain homeostasis in the immune microenvironment. Liver macrophages can be divided into liver-resident phagocytes, known as Kupffer cells (KCs), and bone marrow-derived recruited monocytes (Beattie et al. 2016). Kupffer cells are liver-resident macrophages that play important roles in recognizing and clearing pathogen-associated molecular patterns (PAMPs) and transporting gut bacteria present in portal blood. Furthermore, an inflammatory response is induced by damage-associated molecular patterns (DAMPs) that are released by damaged hepatocytes, which then activate KCs, cytokine/chemokine production and immune cell recruitment (Kazankov et al. 2019; Barreby et al. 2022). Macrophages derived from Ly6 C^low^-infiltrating monocytes exhibit an anti-inflammatory and protective phenotype, while Ly6 C^high^-infiltrating monocytes present a proinflammatory and tissue-detrimental phenotype. The accumulation of proinflammatory macrophages is linked to MASH severity and progression. Anti-inflammatory macrophages can reverse fat accumulation and hepatocyte apoptosis (Xu et al. 2017; Odegaard et al. 2008; Baeck et al. 2014). Therefore, maintaining macrophage homeostasis and effectively regulating the activation of anti-inflammatory macrophages are crucial for controlling the occurrence and development of MASH (Tacke 2017; Horn and Tacke 2024). Thus, controlling the activation of anti-inflammatory macrophages is particularly important for maintaining liver homeostasis and preventing excessive inflammation.

More recently, the secretory phenotype has been defined as the secretion of cytokines, growth factors, proteases, and extracellular vesicles (EVs), which exert paracrine effects on neighboring cells. In addition to soluble cytokines, EVs have been demonstrated to be important mediators of intercellular communication (Pitt et al. 2016). Since these nanosized vesicles surrounded by a lipid bilayer membrane carry proteins, nucleic acids and lipids, EVs can transfer their molecular cargo from donor to recipient cells (Thietart and Rautou 2020). Recent evidence revealed that EVs play an essential regulatory role in the pathogenesis of MASH (Devhare and Ray 2018; Srinivas et al. 2020; Xu et al. 2022), as EVs released from damaged hepatocytes contribute to MASH progression by activating nonparenchymal liver cells, such as hepatic sinusoidal endothelial cells and hepatic stellate cells. Lipid-stimulated hepatocytes secrete TNF-related apoptosis-inducing ligands, which can induce an inflammatory phenotype in mouse bone marrow-derived macrophages (Hirsova et al. 2016). EVs containing miR-128-3p induced HSC activation by inhibiting PPAR-γ (Povero et al. 2015), while hepatocyte-derived MASP1 activates HSCs to promote liver fibrogenesis, which are pivotal therapeutic targets in liver fibrosis (Liu et al. 2023).

Oxysterol-binding proteins, which are high-affinity receptors for oxysterols that function as lipid transport proteins within the endoplasmic reticulum, primarily participate in lipid transport at membrane contact sites. Oxysterol-binding protein homologs constitute a 12-member family in mammals (Pietrangelo and Ridgway 2018; Hammond and Pacheco 2019). Osbpl8 is a member of the ORP family that contains a single C-terminal transmembrane domain that targets the protein to the ER. It is a lipophagy receptor that involved in lipid droplet (LD) biogenesis and degradation, as well as steroid and lipid metabolism and transport (Galmes et al. 2016).

Here, we explored the role of EVs derived from anti-inflammatory BMDMs (M2-BMDMs) in murine MASH. Our results showed that M2-BMDM-derived EVs promote liver injury repair and restore normal liver function in mice with MASH and are correlated with the enrichment of Osbpl8. We demonstrated that Osbpl8 is involved in relieving hepatic steatosis via the IRE1-XBP1 axis. We also describe the effect of macrophage-specific Osbpl8 on ER stress, which contributes to MASH intervention.

## Materials and methods

### Animal models

All animal experiments were approved by the Hangzhou Institute of Medicine (HIM), Chinese Academy of Sciences Animal Care and Use Committee and were performed in accordance with the Institutional Guide for the Care and Use of Laboratory Animals. Male 4- to 6-week-old C57BL/6 J mice were purchased from the Zhejiang Experimental Animal Center. The mice were housed in a temperature-controlled room with a 12-hour light/dark cycle and given water and pelleted chow *ad libitum*. The MASH model was induced by feeding the mice a methionine- and choline-deficient diet (Research Diets, A02082002B).

To knock down Osbpl8 in the liver, mice were injected with AAV2-8-shNC (pAAV-TBG-mCherry-3xFlag-miR30 (NC)-WPRE) or AAV2-8-shOsbpl8 (pAAV-TBG-mCherry-3xFlag-miR30 (Osbpl8)-WPRE) at 2 × 10^11^ v.g. via the tail vein. After one week, all the mice were fed an MCD diet for 3 weeks.

### Mouse primary hepatocyte culture and expansion

Mouse primary hepatocytes were isolated from 6-week-old male C57BL/6 J mice by collagenase perfusion and maintained in DMEM/F12 (Gibco) supplemented with 10% FBS, 1% penicillin/streptomycin and 10 nM dexamethasone. The medium was changed every other day.

### Isolation of bone marrow-derived macrophages (BMDMs)

Macrophages were derived from murine bone marrow as previously described. The isolated bone marrow cells were incubated in Iscove’s Modified Dulbecco’s Medium (IMDM) supplemented with 10% fetal bovine serum, 15% L-929 cell culture supernatant and 10 ng/mL M-CSF and cultured for 5 days to allow differentiation into macrophages; on day 3, the culture medium was replaced with fresh BMDM growth medium. On day 7, the mature BMDMs were cultured without stimulation and were defined as quiescent BMDMs (M0-BMDMs). To induce differentiation into anti-inflammatory BMDMs (M2-BMDMs), mature BMDMs were treated with 10 ng/mL IL-4 and 20 ng/mL IL-13. After 48 h, the supernatant was collected for extraction of extracellular vesicles.

### Isolation and identification of extracellular vesicles

Extracellular vesicles were extracted from the conditioned medium by ultracentrifugation. Briefly, the cell culture medium was centrifuged at 2000 rpm for 15 min, 2000 × g for 15 min, and 12,000 × g for 30 min to remove cell debris and dead cells. Finally, the extracellular vesicles were pelleted by ultracentrifugation at 120,000 × g for 120 min at 4 °C. Exosome sizes and particle numbers were determined by nanoparticle tracking analysis (NTA) with Zeta View (Particle Metrix, Germany). Morphological examination was performed using transmission electron microscopy (TEM) (Joel 2010, Japan). Extracellular vesicle pellets were diluted in fixative (1% cacodylate, 2% glutaraldehyde and 2% paraformaldehyde at pH 7.2–7.4). A few drops of the sample were placed on carbon-coated copper grids and negatively stained with 1% aqueous uranyl acetate. After drying at room temperature, the sample was observed under a transmission electron microscope.

### LC‒MS/MS analysis of EVs

The proteomic profile was generated by NewCore Biotech (Shanghai, China). Twenty microliters of sample was thoroughly mixed with RIPA lysis buffer, followed by low-temperature grinding and sonication in an ice bath for complete lysis. After incubation at 4 °C for 10 min and centrifugation at 12,000 rpm for 15 min at 4 °C, the supernatant was transferred to a new EP tube. The concentration of the extracted protein in the supernatant was quantified using the BCA method. The protein was prepared for subsequent analysis through acetone precipitation, resolubilization, reduction, alkylation, enzymatic hydrolysis, SDC removal, and polypeptide desalting.

The peptides were separated and analyzed using a nano-UPLC (EASY-nLC1200) coupled with a Q Exactive HFX Orbitrap instrument equipped with a nanoelectrospray ionization source. The separation procedure involved the use of a reversed-phase column (100 μm ID × 15 cm, Reprosil-Pur 120 C18 AQ, 1.9 μm) and a gradient program with mobile phases. For data-dependent acquisition (DDA), the Orbitrap analyzer was operated at specified resolutions and parameters.

### Statistical analysis

GraphPad Prism software (version 8.0 for Windows, GraphPad Software, Inc., La Jolla, CA) was used for the statistical analyses. Band intensities in the Western blot images were quantified with ImageJ software. The values are expressed as the means ± SDs of at least three independent experiments. The statistical significance of the differences between two groups was assessed by Student’s t test. For comparisons among multiple groups, statistical significance was evaluated by one-way ANOVA followed by the Student–Newman–Keuls test. In all analyses, *p <* 0.05 indicated statistical significance.

Additional methods are available in the *Supporting Information*.

## Results

### Characteristics of macrophage subpopulations in mice with diet-induced MASH

To investigate the subpopulations of macrophages, flow cytometry analysis was performed to assess the proportion of macrophages in the livers of mice with diet-induced MASH. The liver macrophages were gated on the CD45^+^F4/80^hi^CD11b^int^MHCII^hi^ population; we observed that the numbers of Tim4^+^ KCs were decreased (11.5 ± 1.4145% vs. 4.05 ± 1.202%) and that the numbers of Tim4^−^ monocyte-derived macrophages were increased (84.1 ± 4.815% vs. 94.7 ± 0.565%) in mice with MASH (Fig. S1). Activated liver-resident Kupffer cells and the recruitment of extrahepatic macrophages may play important roles in the initiation and progression of diet-induced liver inflammation during the development of MASH. Although the role of different subsets of macrophages in MASH development has not yet been fully elucidated, it is hypothesized that bone marrow-derived macrophages contribute to inflammatory processes and the early stages of MASH development. Previous studies have shown that cells secrete bioactive molecules such as growth factors, chemokines, cytokines and extracellular vesicles in a paracrine manner and exert immunomodulatory and anti-inflammatory effects on the tissue of interest. Accumulating evidence strongly supports the inference that the effects of bone marrow-derived macrophages are mediated mostly via paracrine mechanisms to regulate the pathophysiological processes of MASH.

### Anti-inflammatory BMDM-EVs reduce lipid toxicity and inhibit hepatic inflammation in MASH mice

First, bone marrow-derived macrophages were differentiated into M0-BMDMs (quiescent) or M2-BMDMs (anti-inflammatory) in vitro. The EVs isolated from M0-BMDMs and M2-BMDMs had diameters between 100 nm and 200 nm (155 ± 19.103 nm vs. 166.4 ± 14.323 nm) and exhibited a regular circular shape (Fig. S2A-B). Nanoflow cytometry analysis revealed significant positive expression of CD63 and CD81 (Fig. S2C). Western blotting revealed TSG101 and CD63 expression, while the expression of the endoplasmic reticulum-specific protein GRP78 and the internal control GAPDH was negative (Fig. S2D).

**Fig. 1:**
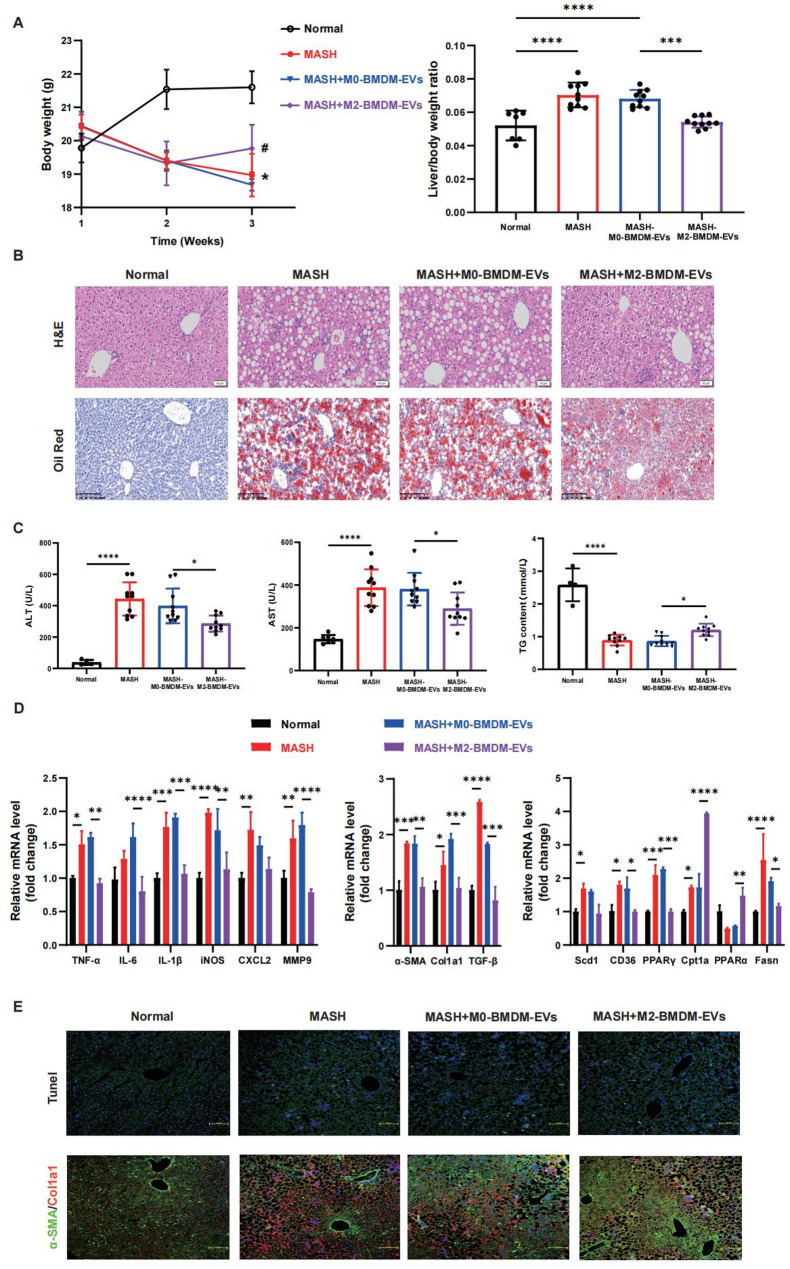
Anti-inflammatory BMDM-EVs reduce lipid toxicity and inhibit hepatic inflammation in MASH mice. A dietary MASH model was established by feeding mice a methionine- and choline-deficient diet for 3 weeks. Mice in the different extracellular vesicle groups were treated with 2 × 10^11^ particles of extracellular vesicles via tail vein injection every 7 days beginning the second week of dietary feeding. Liver samples were collected after 3 weeks of feeding. **A** Liver weight and liver/body weight ratio. **B** Liver sections were stained with H&E and Oil Red O to assess liver injury and steatosis. H&E bar = 50 μm; oil red scale bar = 200 μm. **C** Serum ALT, AST and TG levels were detected using biochemical kits. **D** The expression levels of inflammatory factors, profibrotic genes and genes related to fatty acid metabolism were measured using RT‒qPCR. **E** Hepatocyte apoptosis, macrophage types and liver fibrosis were analyzed using TUNEL and immunofluorescence (bar = 100 μm). * *p <* 0.05; **, *p <* 0.01; ***, *p <* 0.001; ****, *p <* 0.0001 (*n* = 6–8)

We then aimed to elucidate the role of extracellular vesicles derived from BMDMs in a diet-induced mouse model of MASH. Two weeks after the diet was initiated, EVs (2 × 10^11^ particles) were injected into the tail vein every 7 days. The MCD diet significantly inhibited weight gain in MASH mice, while M2-BMDM-EV treatment attenuated weight loss in the third week. However, compared with that in the M0-BMDM-EV-injected group, the liver mass-to-body weight ratio was significantly lower 3 weeks after the injection of M2-BMDM-EVs (Fig. 1A). H&E staining revealed that M2-BMDM-EVs significantly improved steatosis, hepatocyte ballooning, and inflammatory infiltration to prevent MASH. Moreover, Oil Red O staining showed reduced lipid droplet accumulation (Fig. 1B). At the end of the treatment, the serum ALT, AST, and TG levels were markedly decreased compared with those in the control group (Fig. 1C). Moreover, M2-BMDM-EVs suppressed the inflammatory response and fibrosis and effectively improved fatty acid metabolism (Fig. 1D). The expression of a panel of inflammatory markers was measured in circulating blood to evaluate the M1 macrophage response after MASH. Among the 8 inflammatory markers tested, the expression levels of CXCL1 (KC), IL-18, IL-6, TNF-α, IL-12p40, and IL-1β had changed at least 2-fold compared with those in the control group (Fig. 1E). Additionally, M2-BMDM-EV treatment markedly inhibited cell apoptosis and suppressed the expression of liver fibrosis markers, including α-SMA and collagen type I alpha 1 (Col1a1) (Fig. 1F). These results suggested that extracellular vesicles derived from anti-inflammatory BMDMs play a crucial role in controlling inflammatory responses and alleviating hepatotoxicity during MASH.

### Anti-inflammatory BMDM-EVs suppress PA-induced hepatic inflammation and oxidative stress in vitro

**Fig. 2:**
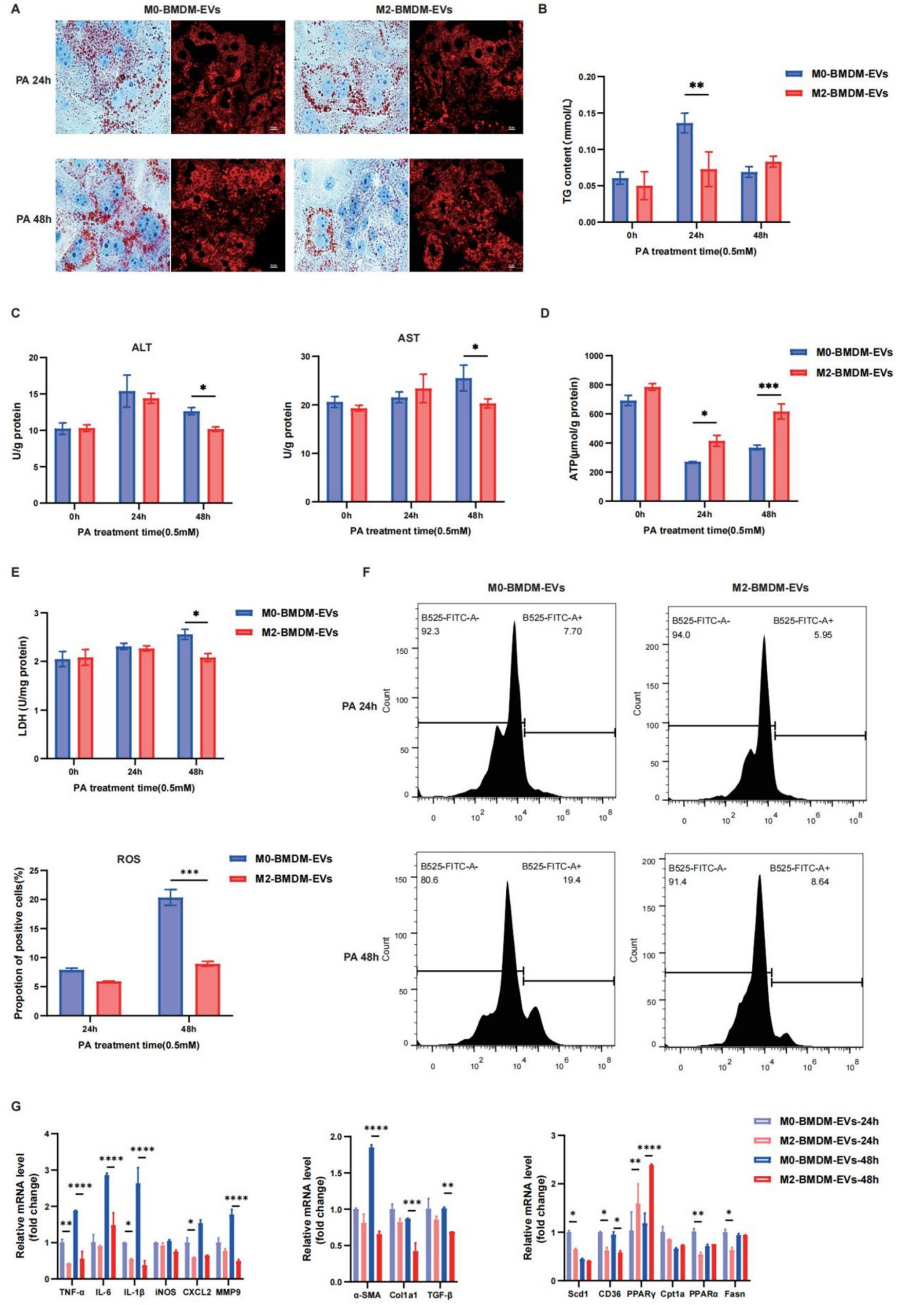
Anti-inflammatory BMDM-EVs suppress PA-induced hepatic inflammation and oxidative stress in vitro. Primary hepatocytes isolated from mice were incubated with palmitic acid (PA, 0.5 mM) to induce lipid droplet accumulation. EVs derived from M0-BMDMs or M2-BMDMs (100 µg/mL) were added after PA induction. **A** Oil red O and Nile red staining shows lipid deposits. Bar = 10 μm. **B** Intracellular TG content. **C** ALT and AST concentrations. **D** ATP production in hepatocytes. **E** LDH release in the cell culture supernatant. **F** Intracellular reactive oxygen species (ROS) levels were detected by flow cytometry and are presented as the percentage of positive cells (%). **G** RT‒qPCR was used to detect the mRNA expression levels of inflammatory factors, profibrotic genes and genes related to fatty acid metabolism; *, *p <* 0.05; **, *p <* 0.01; ***, *p <* 0.001; ****, *p <* 0.0001 (*n* = 3)

Furthermore, in an in vitro model of PA-induced lipid toxicity, we evaluated the effect of BMDM-derived EVs on lipid metabolism and the inflammatory response. Oil Red O and Nile Red staining indicated that PA treatment resulted in a significant accumulation of lipid droplets, while M2-BMDM-EVs inhibited the accumulation of lipid droplets, which indicates an inhibitory effect of M2-BMDM-EVs on fat accumulation (Fig. 2A). The TG concentration was significantly lower after 24 h of M2-BMDM-EV treatment than after M0-BMDM-EV treatment (Fig. 2B). Similarly, the ALT and AST expression levels were significantly lower after 48 h of M2-BMDM-EV treatment (ALT: 12.63 ± 0.4928 vs. 10.18 ± 0.3098 U/g protein; AST: 24.25 ± 2.196 vs. 20.31 ± 0.9092 U/g protein) (Fig. 2C). At 24 and 48 h after M2-BMDM-EV treatment, the ATP concentration gradually increased to a normal level (Fig. 2D). Moreover, PA induced hepatic inflammation and oxidative damage by increasing ROS and LDH levels, and these effects were reversed by treatment with BMDM-EVs (Fig. 2E-F). Moreover, the administration of M2-BMDM-EVs decreased the expression of genes related to inflammation and liver fibrosis and improved fatty acid metabolism (Fig. 2G). Thus, anti-inflammatory BMDM-derived EVs improved liver function by inhibiting the inflammatory response, alleviating liver oxidative stress.

### Proteomic analysis of EVs derived from bone marrow-derived macrophages

**Fig. 3:**
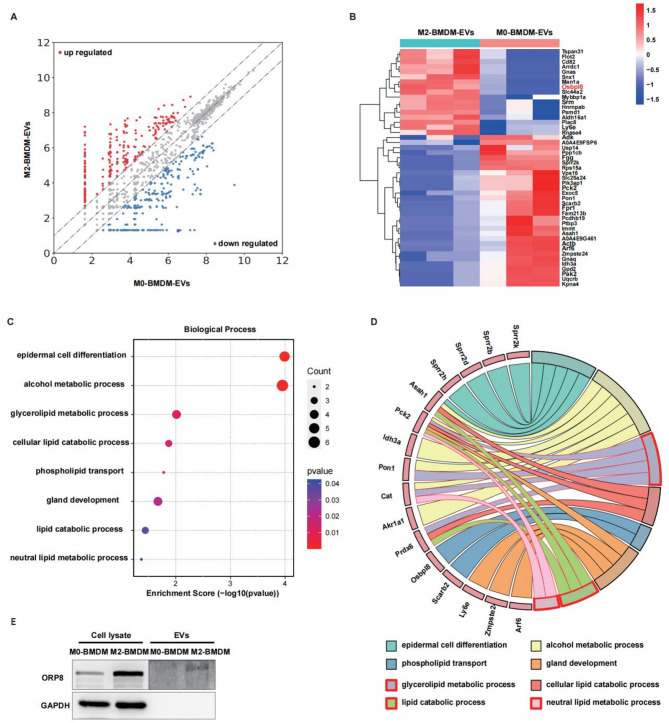
Comparative proteomic analysis of BMDM-derived EVs. Proteomic analysis of the M0-BMDM-EVs and M2-BMDM-EVs was performed using LC–MS/MS analysis. **A** The differentially expressed proteins are displayed in a scatter plot. **B** Heatmap of differential protein enrichment in different types of extracellular vesicles. **C** Bubble plot derived from the GO biological process. **D** Circle plot illustrates the relationship between the genes and GO terms. **E** Osbpl8 protein expression was detected by Western blotting (*n* = 3)

We explored the regulatory mechanism of BMDM-derived EVs using a proteomic analysis to better understand intercellular communication in MASH. Differential expression analysis of the proteomics dataset revealed 17 proteins that were significantly increased in the anti-inflammatory BMDM-derived EVs compared with the control-derived EVs (Fig. 3A-B). Interestingly, the expression of oxysterol-binding protein-related protein 8 (Osbpl8) was enriched in M2-BMDM-EVs; this protein is related to phospholipid transport as well as to several biological processes associated with lipid catabolism and metabolism (Fig. 3C-D). Furthermore, Western blot analysis revealed that Osbpl8 expression was increased in M2-BMDMs and their EVs compared with their corresponding controls (Fig. 3E).

### Osbpl8 attenuates inflammation and lipotoxicity potentially through Endoplasmic reticulum stress regulation

**Fig. 4:**
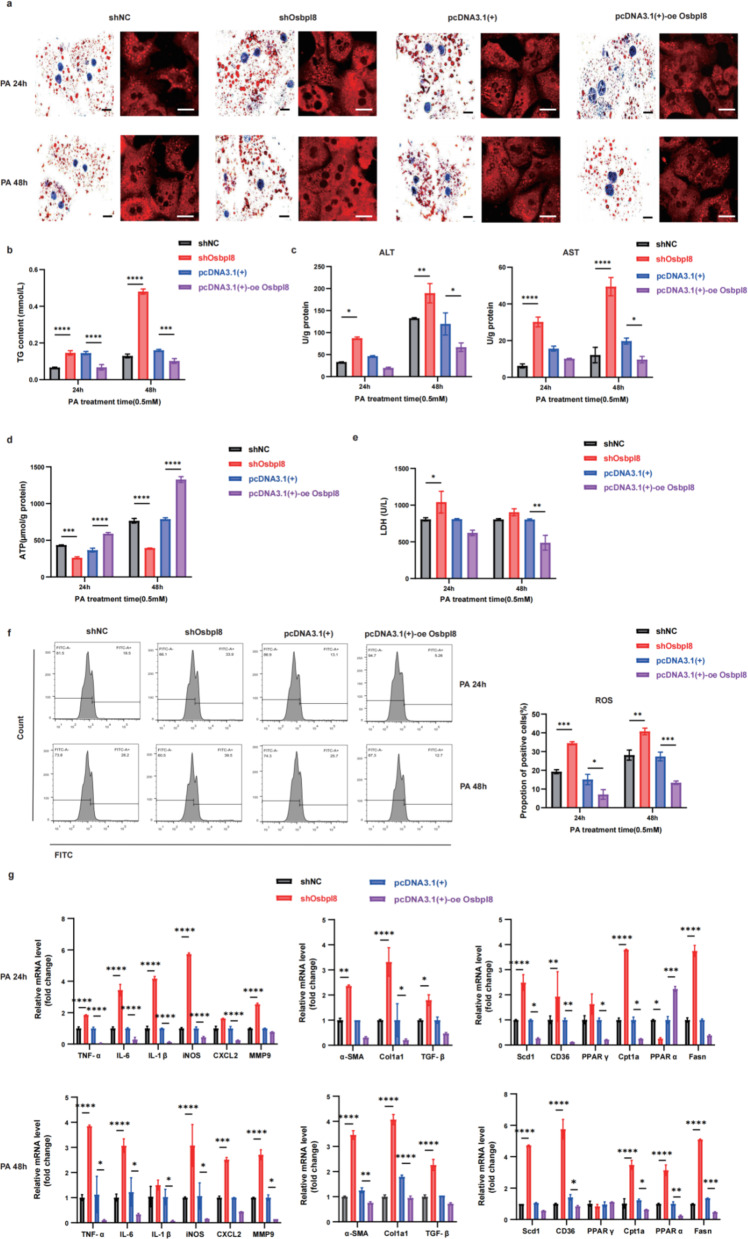
Osbpl8 is involved in the PA-induced hepatocyte inflammatory response and lipid toxicity in vitro. Primary hepatocytes isolated from mice were transfected with an overexpression plasmid or Osbpl8 shRNA to upregulate or downregulate the expression of Osbpl8, respectively. Then, the cells were incubated with palmitic acid (PA, 0.5 mM) to induce lipid droplet accumulation. **A** Oil red O and Nile red staining show lipid deposits. Bar=10 μm. **B** Intracellular TG content. **C** ALT and AST concentrations. **D** ATP production in hepatocytes. **E** LDH release in the cell culture supernatant. **F** Intracellular reactive oxygen species (ROS) levels were detected by flow cytometry and are presented as the percentage of positive cells (%). **G** RT‒qPCR was used to detect the mRNA expression levels of inflammatory factors, profibrotic genes and genes related to fatty acid metabolism; *, *p<*0.05; **, *p<*0.01; ***, *p<*0.001; ****, *p<*0.0001 (*n*=3)

Previous studies reported that lipid transfer proteins ORP5 and ORP8 control LD biogenesis at mitochondria-associated ER membrane (MAM) subdomains, ORP5/8 regulate seipin recruitment to these MAM-LD contacts, and their loss impairs LD biogenesis. Given these findings, we transfected cells with shRNA or overexpression plasmids to downregulate or upregulate Osbpl8 expression, respectively (Fig. S3A-B), to investigate the effects of Osbpl8 on the hepatic cell inflammatory response and lipotoxicity. As shown in Fig. 4A, Osbpl8 decreased intracellular lipid droplet accumulation, as determined by staining with Oil Red O and the lipophilic fluorophore Nile red. Furthermore, Osbpl8 clearly reduced triglyceride accumulation, increased alanine transaminase (ALT) and aspartate transaminase (AST) levels (Fig. 4B-C), accelerated ATP generation (Fig. 4D), and attenuated steatosis, inflammation and oxidative stress (Fig. 4E-F & Fig. S4-S5). In addition, Osbpl8 knockdown promoted anti-inflammatory and fibrotic responses and inhibited fatty acid metabolism (Fig. 4G).

MASLD is characterized by the accumulation of triglycerides and may further progress to MASH if sufficient hepatocyte damage and inflammation occur. Lipid accumulation disrupts the function of the endoplasmic reticulum (ER) in hepatocytes, thereby generating chronic ER stress that interferes with normal cellular function. ER stress has been closely associated with hepatic steatosis, as hepatic lipid overload has been implicated in the initiation of chronic ER stress in steatosis (Ajoolabady et al. 2023; Beaulant et al. 2022). Considering that Osbpl8 is an ER-resident lipid transfer protein involved in lipid metabolism, we sought to investigate whether Osbpl8 prevents MASH progression via ER stress mechanisms.

**Fig. 5:**
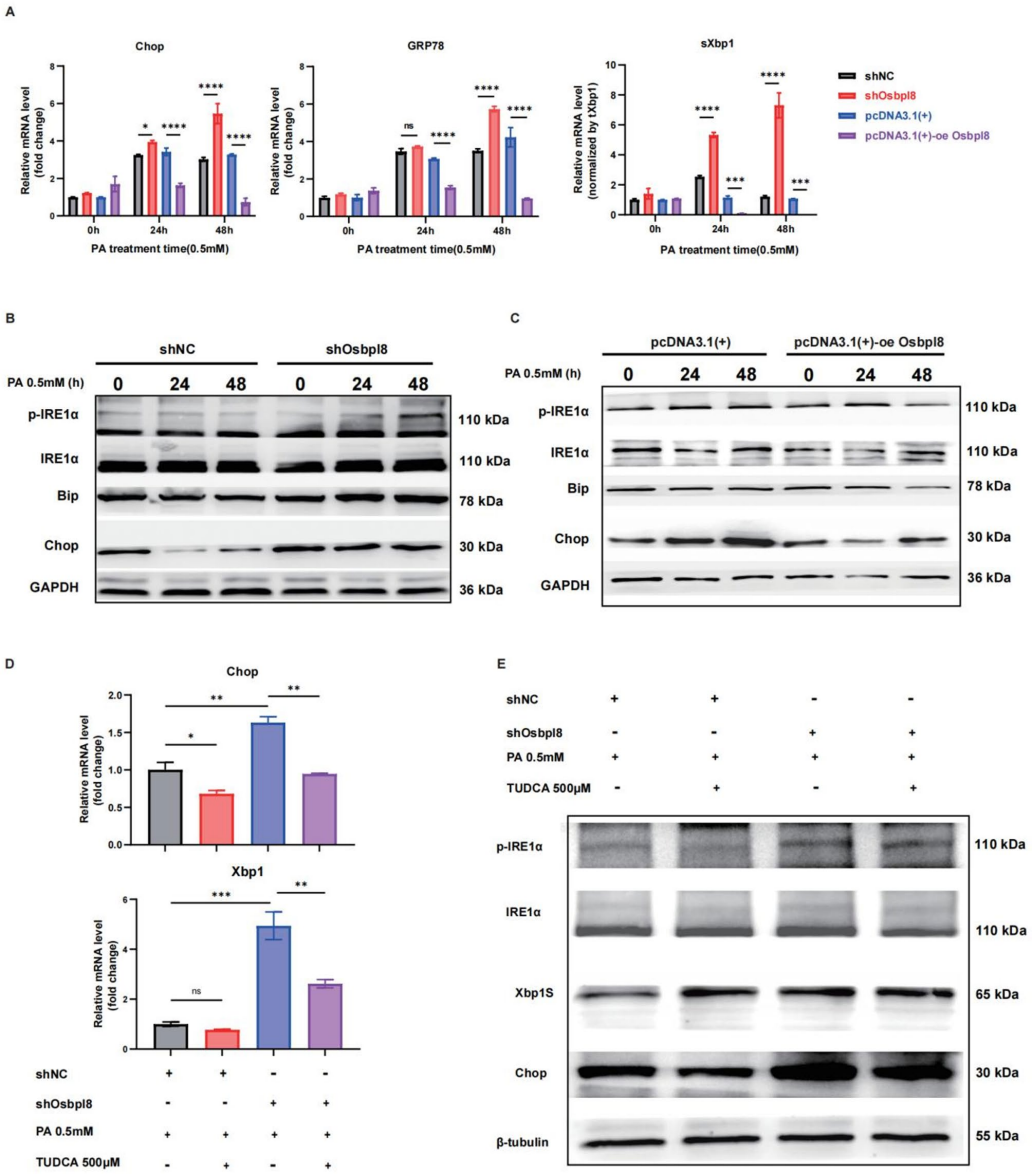
Osbpl8-mediated ER stress mechanism in MASH progression. Primary hepatocytes isolated from mice were transfected with an overexpression plasmid or Osbpl8 shRNA to upregulate or downregulate the expression of Osbpl8, respectively. Then, the cells were incubated with palmitic acid (PA, 0.5 mM) to induce lipid droplet accumulation. Samples were collected after 12 h with or without 500 μM TUDCA. **A** ER stress biomarkers and the expression of UPR-related genes were detected by RT‒qPCR. **B**-**C** The protein expression levels of IRE1α/XBP1 and CHOP were detected by Western blotting. **D** RT‒qPCR and E Western blotting show the effects of TUDCA on the expression of ER stress biomarkers and UPR-related proteins. *, *p *< 0.05; **, *p *< 0.01; ***, *p *< 0.001; ****, *p *< 0.0001 (*n *= 3)

First, we measured the expression of major UPR pathway genes. As shown in Fig. 5A, the expression of ER stress markers, including CHOP, GRP78, and spliced Xbp1, was significantly upregulated in response to Osbpl8 knockdown, while Osbpl8 overexpression effectively inhibited their expression. These results suggested that the potential mechanism by which Osbpl8 inhibits ER stress is related to its regulation of lipid metabolism. Lipotoxicity-induced ER stress can activate inositol-requiring enzyme 1α (IRE1α), and the endoribonuclease activity of IRE1α cleaves X-box binding protein 1 (XBP1) mRNA to generate a spliced variant, XBP1s (Wang et al. 2022). The IRE1α-XBP1 signaling pathway is closely associated with insulin resistance, hepatic steatosis, and inflammation in MASH patients (Wang et al. 2022; An et al. 2024). Western blotting revealed that Osbpl8 attenuates ER stress by inhibiting the phosphorylation of IRE1α and reducing CHOP expression (Fig. 5B-C & S6A-B), and pretreatment with the ER stress inhibitor tauroursodeoxycholic acid (TUDCA) reversed these effects (Fig. 5D-E & S6C). Overall, our data strongly suggested that Osbpl8 alleviates inflammation and remodels lipid metabolism by inhibiting excessive ER stress in the setting of PA-induced hepatic lipotoxicity.

### Osbpl8 effectively inhibits the progression of MASH in vivo

**Fig. 6:**
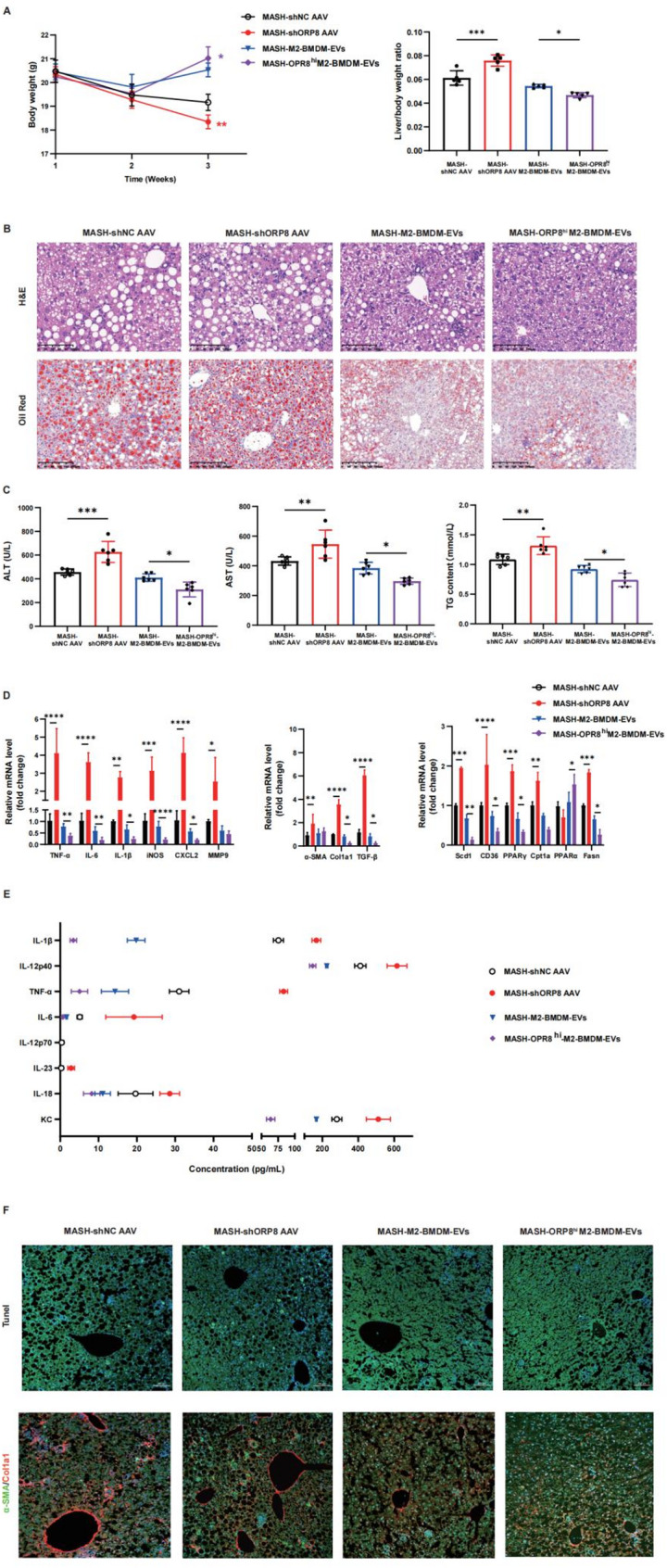
Exploring the therapeutic potential of Osbpl8 in targeting inflammation and lipotoxicity in MASH. The dietary MASH model was established by feeding mice a high-trans-fat/cholesterol diet for at least 3 weeks. Adeno-associated virus (AAV)2/9 (1×1012 vg/kg) containing Osbpl8-specific shRNA was injected into the tail vein of the mice to establish a liver-specific Osbpl8 gene knockdown model. Alternatively, the mice were treated with M2-BMDM-EVs or OSBPL8-enriched M2-BMDM-EVs (2×1011 particles of extracellular vesicles) via tail vein injection every 7 days beginning the second week of dietary feeding. Liver samples were collected after 3 weeks of feeding. **A** Liver weight and liver/body weight ratio. **B** Liver sections were stained with H&E and Oil Red O to assess liver injury and steatosis. H&E bar=100 μm; oil red scale bar=200 μm. **C** Serum ALT, AST and TG levels were detected via biochemical kits. **D** The expression levels of inflammatory factors, profibrotic genes and genes related to fatty acid metabolism were measured using RT‒qPCR. **E** The expression levels of M1 macrophage markers in serum were detected by flow cytometry. **F** Hepatocyte apoptosis, macrophage types and liver fibrosis were analyzed by TUNEL and immunofluorescence (bar=100 μm). * *p *< 0.05; **, *p *< 0.01; ***, *p *< 0.001; ****, *p *< 0.0001 (*n *= 6-8)

To further determine whether Osbpl8 attenuates MASH in vivo, we injected an adenovirus expressing Osbpl8-specific shRNA (1 × 10^12^ vg/kg) or Osbpl8-enriched M2-BMDM-derived EVs (2 × 10^11^ particles/mouse) into the tail vein to regulate Osbpl8 expression. We found that Osbpl8 reversed body weight loss in mice with diet-induced MASH (Fig. 6A). H&E and Oil Red O staining revealed that, compared with mice in the control group, Osbpl8-knockdown mice exhibited severe hepatic steatosis, hepatocyte ballooning, and neutrophil infiltration, while the administration of Osbpl8-enriched M2-BMDM-EVs significantly improved hepatic steatosis and inflammation (Fig. 6 & S7). The ShOsbpl8-AAV group exhibited significant increases in the serum ALT, AST, and TG levels, whereas Osbpl8-enriched M2-BMDM-EV injection ameliorated liver injury in MASH mice (Fig. 6C). Furthermore, we evaluated inflammatory responses, profibrotic genes, and fatty acid metabolism capacity. Osbpl8 knockdown promoted inflammation, fibrosis, and dysregulated fatty acid metabolism (Fig. 6D). Expression of the M1 macrophage markers CXCL1 (KC), IL-6, TNF-α, IL-12p40, and IL-1β was significantly decreased in the Osbpl8-enriched M2-BMDM-EV group (Fig. 6E). Additionally, TUNEL staining revealed that Osbpl8-enriched M2-BMDM-EVs significantly inhibited hepatocyte apoptosis. Immunofluorescence staining revealed that treatment with Osbpl8-enriched M2-BMDM-EVs promoted anti-inflammatory and antilipotoxic effects (Fig. 6F), which demonstrates the important role of Osbpl8 in inhibiting inflammatory reactions, reducing hepatic lipotoxicity, regulating lipid metabolism homeostasis, and restoring normal liver function. These findings could lead to the identification of a novel therapeutic target for the clinical treatment of MASH.

## Discussion

MASLD is a complex, multifactorial disease that can progress to severe liver disease. Although MASH develops within the background of a metabolic disorder, it has strong immunoinflammatory effects (Schuster et al. 2018). A complex network of immune cells, fibrosis, cirrhosis and HCC promotes the transition from simple steatosis to MASH (Pfister et al. 2021). Therefore, exploring the composition and function of immune cells is highly important for understanding their role in MASH development. This study helps to elucidate the potential mechanism of diet-induced MASH and identifies a novel therapeutic target. In this study, we demonstrated that anti-inflammatory bone marrow-derived macrophages inhibit inflammation and attenuate lipotoxicity by secreting Osbpl8-enriched extracellular vesicles and restoring normal liver function by regulating endoplasmic reticulum stress-related mechanisms in diet-induced MASH, which provides new ideas for the treatment of early-stage MASH.

Liver macrophages, which primarily include Kupffer cells and monocyte-derived macrophages (Mo-MFs), are characterized by high diversity and plasticity and play vital roles in the regulation of inflammation and fibrogenesis in MASH. Abundant evidence indicates that macrophages are involved in the early stages of steatosis and inflammation that occur during MASH (Dasgupta et al. 2020; Wang et al. 2024). In one study, in an MCD diet-induced MASH model, the number of KCs was decreased, while the level of inflammatory infiltration by Mo-MFs in the liver was robust (Du et al. 2019). Additionally, high-fat diet (HFD)-fed mice were shown to have an increased number of liver macrophages with a preponderance of proinflammatory cytokine production. Anti-inflammatory macrophages are mainly associated with the remission of MASH and improvements in insulin resistance. These cells are known to secrete anti-inflammatory cytokines, such as arginase-1 and IL-10, which resolve inflammation and promote tissue repair. In our study, we found that monocyte-derived macrophages were recruited to the liver during MASH and that they could differentiate into cells with proinflammatory phenotypes that promote MASH progression.

To our knowledge, macrophages are crucial mediators of MASH, and the specific regulatory mechanisms involved require further exploration. Increasing numbers of studies have indicated that exosomes are important mediators of cell-cell communication (Balaphas et al. 2019). To investigate the biological functions of BMDM-derived EVs, we systematically analyzed the EVs using label-free relative quantitative proteomics. GO enrichment analysis revealed the principal pathways affected and suggested that M2-BMDM-EVs are related mainly to metabolic processes and phospholipid transport, while the KEGG pathway enrichment analysis revealed that the Nod-like receptor signaling pathway, which is intimately involved in ER stress, is an important pathway regulated by Tregs. It has been reported that the ORP family protein ORP8, which is encoded by the Osbpl8 gene, contains a trans-membrane segment at the C-terminus that is specifically localized at the ER (Chung et al. 2023). Additionally, ORP8 is highly enriched in anti-inflammatory BMDMs. Yan et al. indicated that ORP8 decreases cholesterol efflux in macrophages by suppressing ABCA1 expression, which suggests that ORP8 may play a role in the development of atherosclerotic lesions (Yan et al. 2008). Studies have reported that ORP8 inhibits cell migration by interacting with the nuclear pore protein Nup62, which regulates the cell cycle through interaction with ASTIN/SPAG519 and transports HPS at endoplasmic reticulum-membrane contact sites (Beaslas et al. 2012). Our findings provide evidence that Osbpl8 significantly reduces lipid accumulation in the liver, inhibits the inflammatory response and lipotoxicity by regulating lipid metabolic homeostasis, and promotes the restoration of liver function; thus, Osbpl8 is an attractive target for developing therapeutic drugs for MASH.

Mammalian oxysterol-binding protein-related protein (ORP) functions as a sterol sensor that regulates numerous cellular functions, including sterol and neutral lipid metabolism, vesicle transport, and cell signaling (Ghai et al. 2017; Pu et al. 2023; Diercks et al. 2024). ORP8 plays a role in the transportation of phosphatidylserine to ER-plasma membrane contact sites, which indicates a potential role in the regulation of ER function. Aberrant changes in lipids in hepatocytes during hepatic steatosis can directly trigger chronic ER stress in the liver Lipid-induced ER stress involves two primary mechanisms: changes in membrane fluidity and perturbation of Ca^2+^ homeostasis. Lipid overload affects the membrane lipid composition, as indicated by an increased phosphatidylcholine/phosphatidylethanolamine ratio, which alters membrane fluidity. The other is known as the unfolded protein response (UPR), which is an intracellular stress signaling cascade that protects cells from stress caused by the accumulation of unfolded or misfolded proteins and is highly sensitive to changes in intracellular homeostasis (Lebeaupin et al. 2018). Our in vitro and in vivo results supported the lipogenic role of Osbpl8 in relieving hepatic steatosis via the IRE1-XBP1 axis.

## Conclusion

In summary, our results show that extracellular vesicles derived from anti-inflammatory BMDMs regulate inflammatory responses and alleviate lipotoxicity by transporting Osbpl8, promoting liver injury repair and restoring normal liver function through endoplasmic reticulum stress-related mechanisms in murine MASH. These findings illustrate a new dialog between macrophages and hepatocytes in the immune microenvironment, which represents a novel mechanism of macrophage homoeostasis in MASH. These findings highlight Osbpl8 as a promising therapeutic target for MASH and suggest new strategies for early intervention in MASH.

## Supplementary Information

Supplementary Material 1.

## Data Availability

No datasets were generated or analysed during the current study.

## Abbreviations

BMDMs: Bone marrow-derived macrophages
DAMPs: Damage-associated molecular patterns
ER: Endoplasmic reticulum
EVs: Extracellular vesicles
KCs: Kupffer cells
LDs: Lipid droplets
MASH: Metabolic dysfunction-associated steatohepatitis
MASLD: Metabolic dysfunction-associated steatotic liver disease
NTA: Nanoparticle tracking analysis
NAFLD: Nonalcoholic fatty liver disease
NASH: Nonalcoholic steatohepatitis
ORP8/Osbpl8: Oxysterol-binding protein-related protein 8
PA: Palmitic acid
PAMPs: Pathogen-associated molecular patterns
TUDCA: Tauroursodeoxycholic acid
