# Correction: BMDM-derived ORP8 suppresses lipotoxicity and inflammation by relieving Endoplasmic reticulum stress in mice with MASH

**DOI:** 10.1186/s10020-025-01332-0

**Published:** 2025-07-28

**Authors:** Yi Chen, Kangjie Xie, Caiyang Chen, Xihui Wang, Chenchen Ma, Zhangxiang Huang, Yingfu Jiao, Weifeng Yu

**Affiliations:** 1 Department of Anesthesiology, Renji Hospital, Jiaotong University School of Medicine, No. 160, Pujian Road, Pudong New District, Shanghai, 200217 China; 2 Key Laboratory of Anesthesiology, Shanghai Jiao Tong University, Ministry of Education, Shanghai, 200217 China; 3 Department of Anesthesiology, The First Affiliated Hospital of Wenzhou Medical University, Wenzhou, 310022 Zhejiang China; 4 Department of Anesthesiology, Zhejiang Cancer Hospital, Hangzhou Institute of Medicine (HIM), Chinese Academy of Sciences, Hangzhou, 310022 Zhejiang China; 5 Department of Pain Management, The First Affiliated Hospital of Kunming Medical University, Kunming, Yunnan, 650032 China

**Correction: Molecular Medicine 31, 213 (2025)**

**https://doi.org/10.1186/s10020-025-01275-6**

In this article (Chen et al. 2025), the legend of Figs. 5E and 6E and F were omitted in Figs. 5 and 6; the figure should have appeared as shown below.

Incorrect figure Caption:

**Fig. 5** Osbpl8-mediated ER stress mechanism in MASH progression. Primary hepatocytes isolated from mice were transfected with an overexpression plasmid or Osbpl8 shRNA to upregulate or downregulate the expression of Osbpl8, respectively. Then, the cells were incubated with palmitic acid (PA, 0.5 mM) to induce lipid droplet accumulation. Samples were collected after 12 h with or without 500 µM TUDCA. **A** ER stress biomarkers and the expression of UPR-related genes were detected by RT‒qPCR. **B** The protein expression levels of IRE1α/XBP1 and CHOP were detected by Western blotting. C RT‒qPCR and D Western blotting show the effects of TUDCA on the expression of ER stress biomarkers and UPR-related proteins. *, *p* < 0.05; **, *p* < 0.01; ***, *p* < 0.001; ****, *p* < 0.0001 (*n* = 3)

**Fig. 6** Exploring the therapeutic potential of Osbpl8 in targeting inflammation and lipotoxicity in MASH. The dietary MASH model was established by feeding mice a high-trans-fat/cholesterol diet for at least 3 weeks. Adeno-associated virus (AAV)2/9 (1 × 1012 vg/kg) containing Osbpl8-specific shRNA was injected into the tail vein of the mice to establish a liver-specific Osbpl8 gene knockdown model. Alternatively, the mice were treated with M2-BMDM-EVs or OSBPL8-enriched M2-BMDM-EVs (2 × 1011 particles of extracellular vesicles) via tail vein injection every 7 days beginning the second week of dietary feeding. Liver samples were collected after 3 weeks of feeding. **A** Liver weight and liver/body weight ratio. **B** Liver sections were stained with H&E and Oil Red O to assess liver injury and steatosis. H&E bar = 100 μm; oil red scale bar = 200 μm. **C** Serum ALT, AST and TG levels were detected via biochemical kits. **D** The expression levels of inflammatory factors, profibrotic genes and genes related to fatty acid metabolism were measured using RT‒qPCR. E Hepatocyte apoptosis, macrophage types and liver fibrosis were analyzed by TUNEL and immunofluorescence (bar = 100 μm). * *p* < 0.05; **, *p* < 0.01; ***, *p* < 0.001; ****, *p* < 0.0001 (*n* = 6–8)

Corrected Caption:

**Fig. 5:**
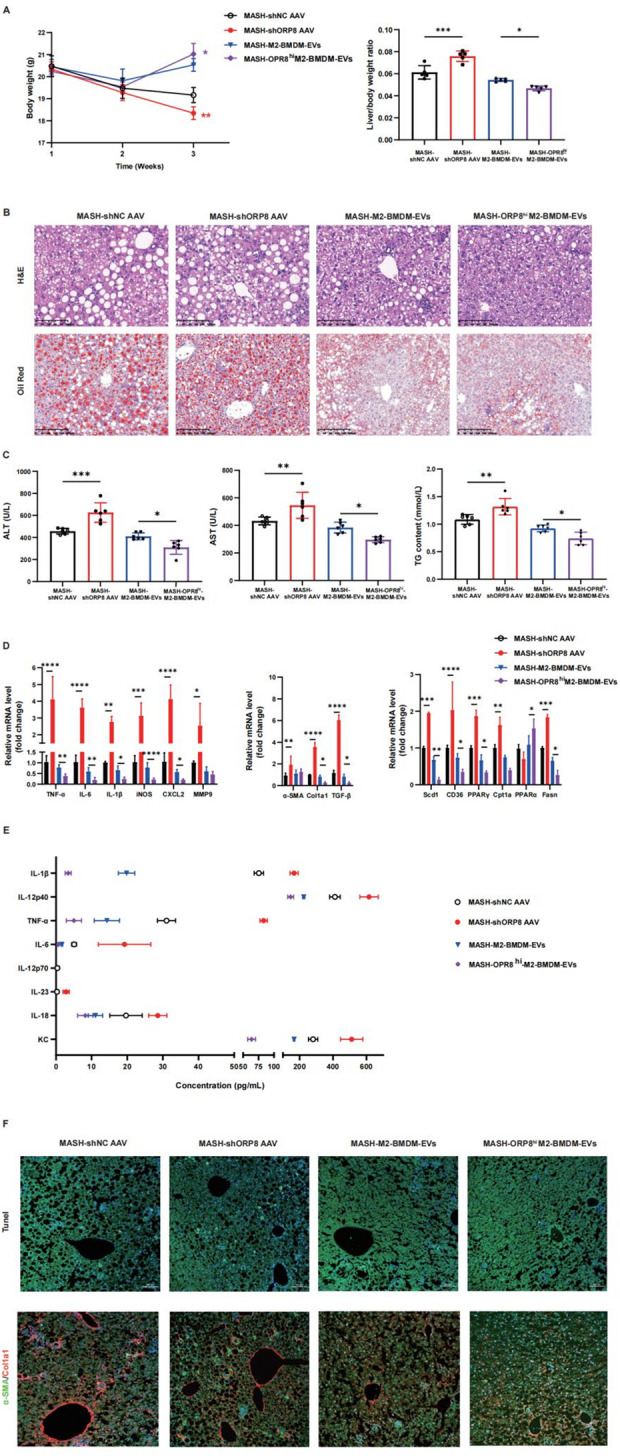
Osbpl8-mediated ER stress mechanism in MASH progression. Primary hepatocytes isolated from mice were transfected with an overexpression plasmid or Osbpl8 shRNA to upregulate or downregulate the expression of Osbpl8, respectively. Then, the cells were incubated with palmitic acid (PA, 0.5 mM) to induce lipid droplet accumulation. Samples were collected after 12 h with or without 500 µM TUDCA. **A** ER stress biomarkers and the expression of UPR-related genes were detected by RT‒qPCR. **B**-**C** The protein expression levels of IRE1α/XBP1 and CHOP were detected by Western blotting. **D** RT‒qPCR and **E** Western blotting show the effects of TUDCA on the expression of ER stress biomarkers and UPR-related proteins. *, *p* < 0.05; **, *p* < 0.01; ***, *p* < 0.001; ****, *p* < 0.0001 (*n* = 3)

**Fig. 6:**
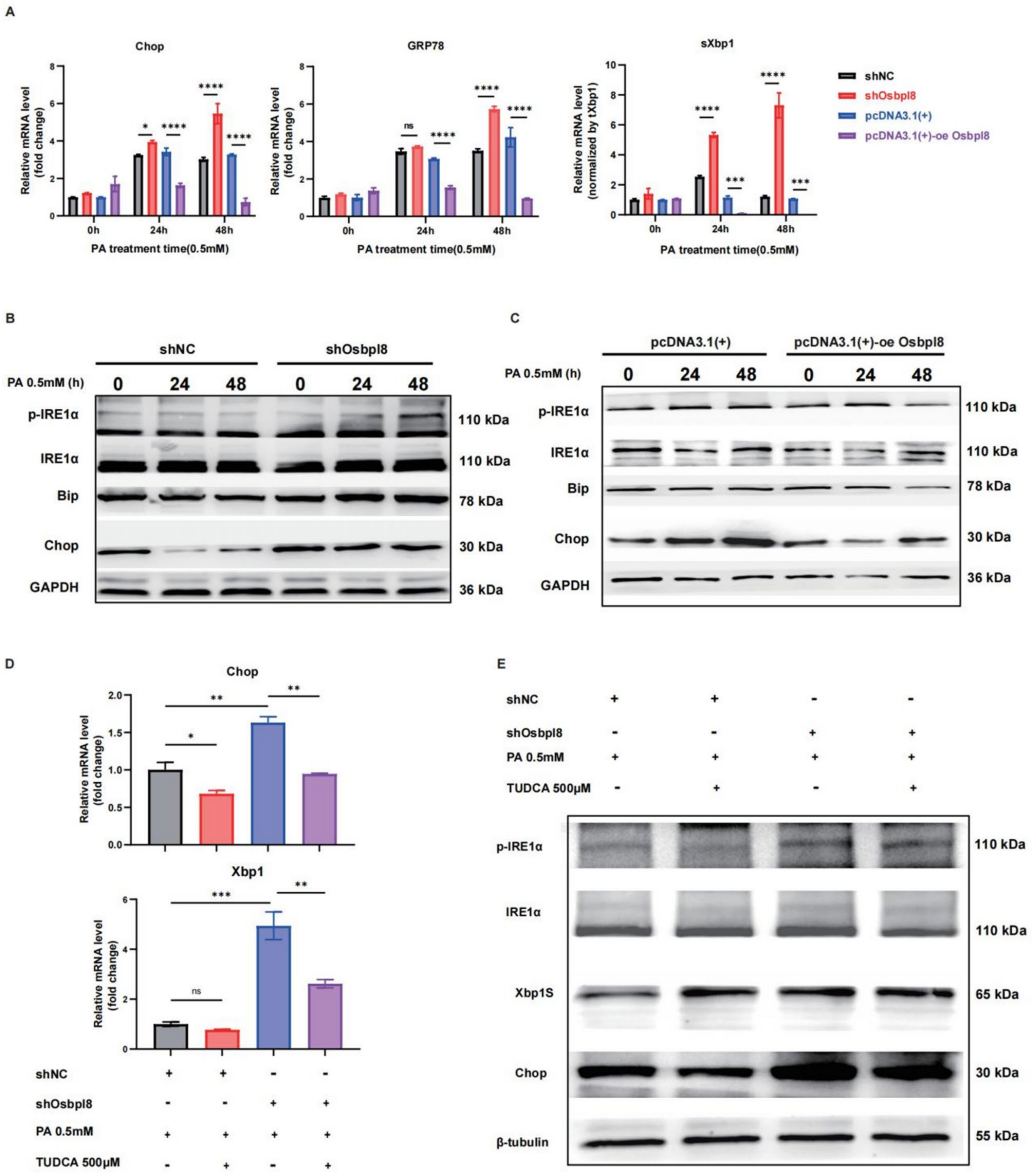
Exploring the therapeutic potential of Osbpl8 in targeting inflammation and lipotoxicity in MASH. The dietary MASH model was established by feeding mice a high-trans-fat/cholesterol diet for at least 3 weeks. Adeno-associated virus (AAV)2/9 (1 × 1012 vg/kg) containing Osbpl8-specific shRNA was injected into the tail vein of the mice to establish a liver-specific Osbpl8 gene knockdown model. Alternatively, the mice were treated with M2-BMDM-EVs or OSBPL8-enriched M2-BMDM-EVs (2 × 1011 particles of extracellular vesicles) via tail vein injection every 7 days beginning the second week of dietary feeding. Liver samples were collected after 3 weeks of feeding. **A** Liver weight and liver/body weight ratio. **B** Liver sections were stained with H&E and Oil Red O to assess liver injury and steatosis. H&E bar = 100 μm; oil red scale bar = 200 μm. **C** Serum ALT, AST and TG levels were detected via biochemical kits. **D** The expression levels of inflammatory factors, profibrotic genes and genes related to fatty acid metabolism were measured using RT‒qPCR. **E** The expression levels of M1 macrophage markers in serum were detected by flow cytometry. **F** Hepatocyte apoptosis, macrophage types and liver fibrosis were analyzed by TUNEL and immunofluorescence (bar = 100 μm). * *p* < 0.05; **, *p* < 0.01; ***, *p* < 0.001; ****, *p* < 0.0001 (*n* = 6–8)
