# Supplementary material for: BMDM-derived ORP8 suppresses lipotoxicity and inflammation by relieving endoplasmic reticulum stress in mice with MASH
Source: Mol Med. 2025 May 30;31:213. doi: 10.1186/s10020-025-01275-6 (PMC12123767; doi:10.1186/s10020-025-01275-6)
Supplement: Supplementary file 1 — Supplementary Material 1. [file 10020_2025_1275_MOESM1_ESM.docx]

**SUPPLEMENTARY MATERIALS**

**BMDM-derived ORP8 Suppresses Lipotoxicity and Inflammation by Relieving Endoplasmic Reticulum Stress in Mice with MASH**

Yi Chen^1,2,*^, Kangjie Xie^3,*^, Caiyang Chen^1,2,*^, Xihui Wang^3^, Chenchen Ma^3^, Zhangxiang Huang^4,✉,^ Yingfu Jiao^1,2,✉^, Weifeng Yu^1,2,✉^

**1. Additional file 1: Supplementary materials and methods**

**2. Supplementary figure**

**2.1 Additional file 2: Supplementary Fig. S1**

**2.2 Additional file 3: Supplementary Fig. S2**

**2.3 Additional file 4: Supplementary Fig. S3**

**2.3 Additional file 4: Supplementary Fig. S4**

**2.3 Additional file 4: Supplementary Fig. S5**

**2.3 Additional file 4: Supplementary Fig. S6**

**2.3 Additional file 4: Supplementary Fig. S7**

**3. Supplementary table**

**3.1 Additional file 7: Supplementary Table S1**

**3.2 Additional file 8: Supplementary Table. S2**

**Additional file 1**

**SUPPLEMENTARY MATERIALS AND METHODS**

**Cell treatment**

For experiments analyzing the function of EVs, we isolated and cultured primary murine hepatocytes until they reached confluence. The medium was changed, and 100 μg/mL EVs were added. After the cells were stimulated with PA (0.5 mM) for 24 h and 48 h, we collected the cells for further detection.

For cell transfection experiments, plasmids were transfected into cells using a Lipofectamine 3000 transfection kit. Osbpl8-overexpressing or -knockdown cells were generated via transfection with Ospbl8-pcDNA3.1(+)-C-Myc plasmid or Osbpl8-pLKO.1-copGFP-PURO shRNA (Genescript, Nanjing, China).

**Flow cytometry**

Specific surface markers expressed on EVs were assessed by flow cytometry. EVs were resuspended in PBS and stained with antibodies against CD63 and CD81 (BD Pharmingen). Dead cells were excluded during the flow cytometry analysis, and gating was performed based on isotype controls. The stained cells were analyzed using a Flow NanoAnalyzer (Fuliu, Xiamen, China).

**Real-time quantitative PCR (RT‒qPCR)**

Total RNA was extracted from liver tissues and primary human hepatocytes using TRIzol reagent (Invitrogen Life Technologies) according to the manufacturer’s instructions. RNA transcripts were quantified with brilliant SYBR Green qPCR (Takara, Kyoto, Japan) using a Roche Light-Cycler 4800II real-time PCR system (Roche). The primers used are listed in Table S1. All the data were analyzed using 18S rRNA as an internal control. The relative number of copies of the target gene was determined using the 2^-^*^ΔΔ^*^Ct^ method.

**Western blotting**

Liver samples and cells were harvested and lysed using RIPA buffer supplemented with PMSF and a complete protease inhibitor cocktail. The samples were mixed continuously by inversion in a rotating agitator at 4°C for 1 h and then centrifuged at 12000 × g and 4°C for 15 min to pellet insoluble material. The supernatants were collected, and the protein concentrations were determined using a BCA protein assay kit (Pierce, USA). Briefly, the proteins were separated by SDS‒PAGE and transferred to polyvinylidene fluoride membranes (Hybond-P, GE Healthcare, Singapore). The membranes were incubated for 1 h in 5% nonfat milk and Tris-buffered saline with Tween-20 and then incubated overnight at 4°C with primary antibodies. The membranes were then incubated with HRP-conjugated secondary antibodies for 1 h at room temperature. The proteins were visualized with an enhanced chemiluminescence kit (Pierce, USA) on a ^ChemiDocTM^ XRS^+^ system (Bio-Rad). The antibody references used are listed in Table S2.

**Histology and lipid staining**

Liver tissues were fixed immediately with 4% formaldehyde for histological analysis. Sections (4 µm thick) were prepared from paraffin-embedded tissues and subjected to either hematoxylin and eosin (H&E) staining or immunostaining. Images of H&E-stained sections were captured under a microscope (Biozero BZ-9000 Series; Keyence, Osaka, Japan). The sections were also stained for oil red O. The cells were washed with 60% isopropanol and stained with diluted oil red O working solution at room temperature for 30 min. Then, the cells were rinsed in running water. The cells were restrained in hematoxylin solution for 1–2 min and washed with running water. The cells were photographed and recorded by a microscope (BX53; Olympus, Tokyo, Japan). Histological scoring was performed by two specialists blinded to the experimental design and data. Scoring ranges were as follows: Degree of steatosis (0-3), lobular inflammation (0-3), hepatocyte ballooning (0-2).

The cells were fixed with 4% paraformaldehyde and then stained with 1 μM Nile red for 45 min at room temperature. The fluorescence of Nile red was determined by confocal microscopy (Nikon A1R-HD25) using 540-nm excitation and 590-nm emission wavelengths.

**Immunofluorescence staining**

Liver samples were collected, fixed with 4% formaldehyde and then cryoprotected in 30% sucrose for 48 h. Liver tissues were cut in the coronal plane on a cryostat (Leica) at 8 μm and processed for immunofluorescence staining. The liver sections were permeabilized in 0.3% Triton X-100 (Sigma) for 10 min, blocked in PBS containing 5% BSA for 1 h and incubated overnight with the following primary antibodies at 4°C. The sections were then washed with PBST and incubated with the appropriate secondary antibodies for 1 h at 37°C. The slides were covered with glass slide covers supplemented with mounting media containing 4,6-diamidino-2-phenylindole (DAPI). Images were acquired with a confocal microscope (Nikon A1R-HD25), and the images were analyzed using ImageJ (version 6.0).

**ALT and AST**

Blood was collected from the mice, and serum was extracted from the blood. The serum ALT and AST levels were measured with the following colorimetric assays according to the manufacturer’s recommendations: Liquid ALT (SGPT) and Liquid AST (SGOT) (Pointe Scientific).

**ATP measurement**

**The** ATP concentration was determined using an ATP determination kit (Invitrogen, Oregon, USA). The ATP concentration was determined according to the manufacturer’s protocol. Luminescence was measured in a TECAN Spark plate reader and normalized to the total protein content.

**ROS analysis**

To detect intracellular ROS production, the cells were incubated with 10 μM DCFH-DA for 30 min at 37°C. The assay is based on the ability of DCFH-DA to enter cells through hydrophobic regions. Once inside, the dye is cleaved by intracellular esterases to yield 2’-7’-dichlorofluorescin (DCFH). Because of its polar nature, DCFH is trapped intracellularly and can be oxidized to fluorescent dichlorofluorescin (DCF) by cellular oxidants. The fluorescence intensity of DCF (excitation: 488 nm; emission: 527 nm) was measured by flow cytometry.

**LDH release**

Lactate dehydrogenase (LDH) release in the supernatants of cultured cells and cell extracts was determined using an enzyme activity assay kit (Cell Signaling Technology) according to the manufacturer’s instructions.

**2. Supplementary figure**

**
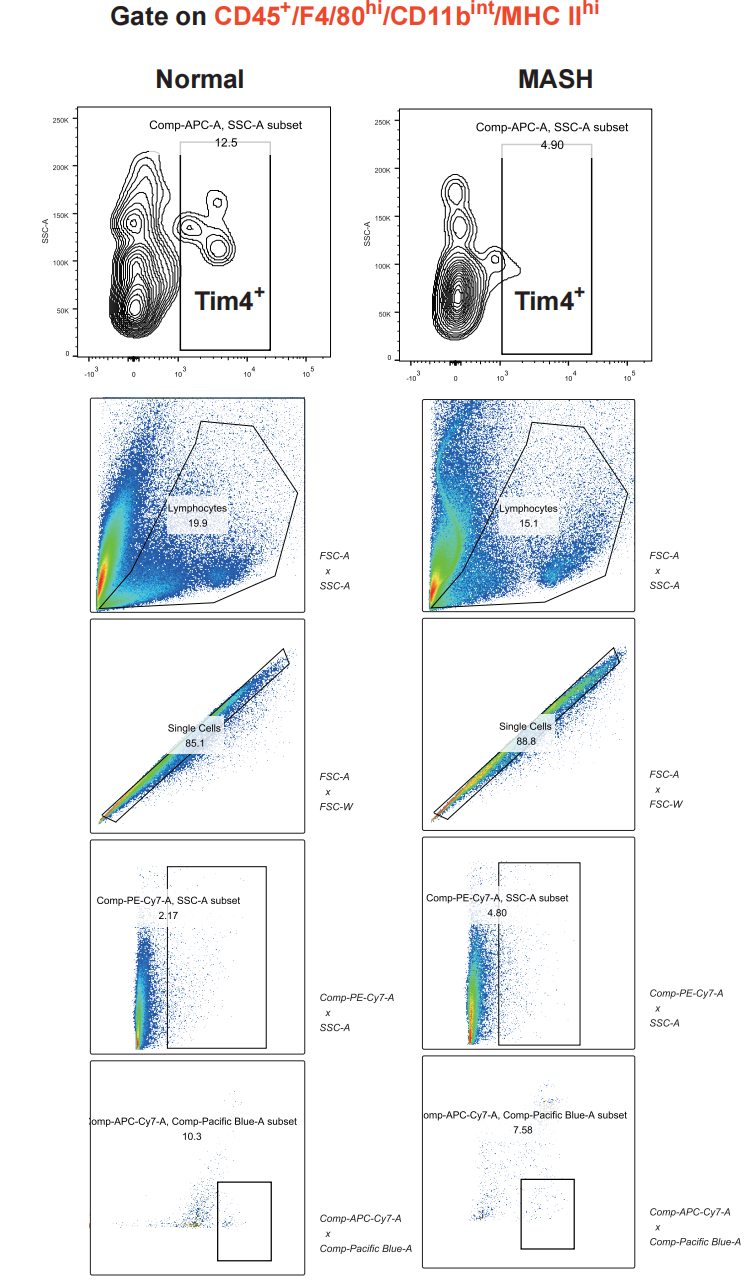
Fig. S1 Distribution characteristics of macrophage subsets in MASH livers via flow cytometry.**


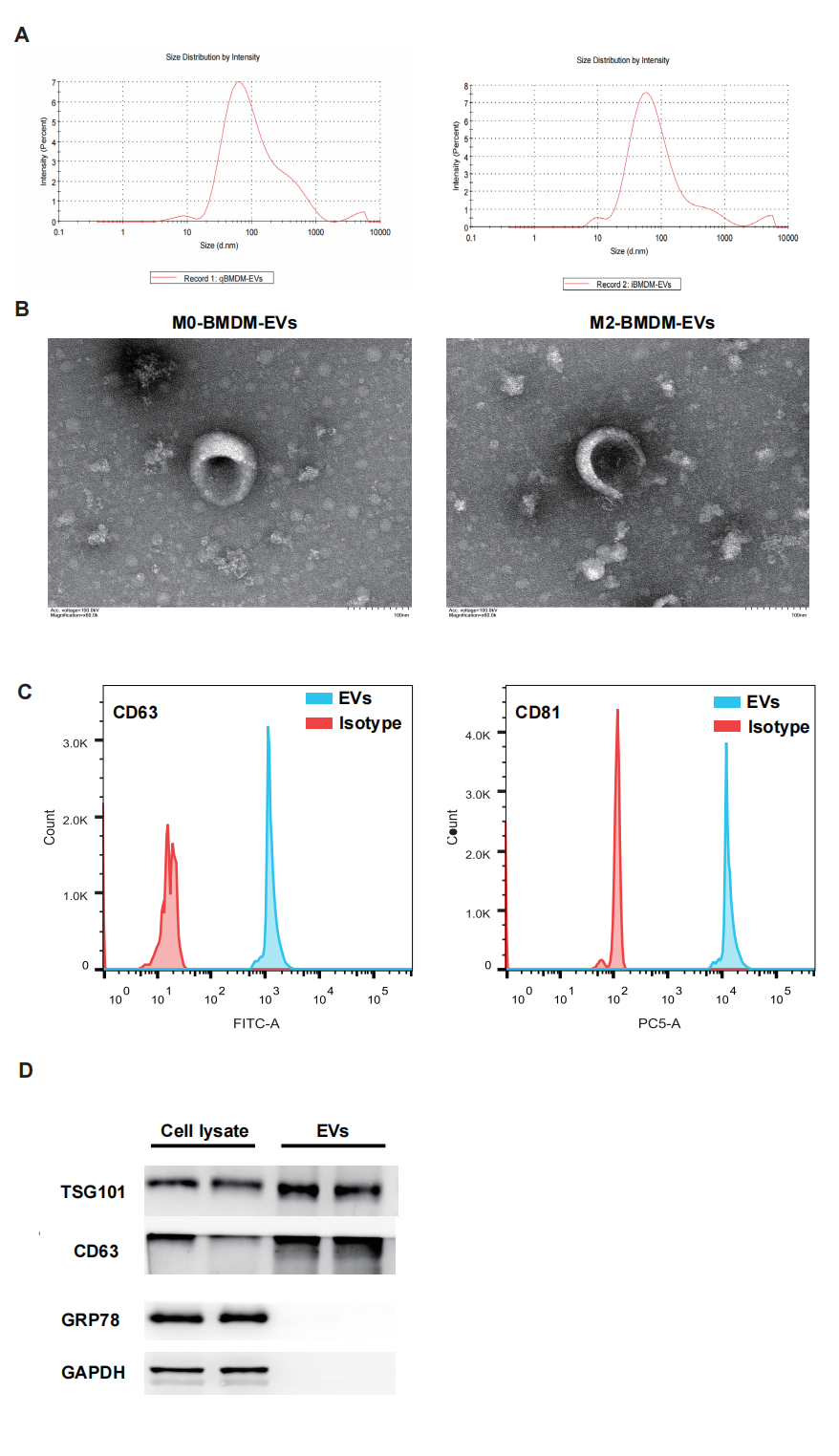


**Fig. S2 Identification of BMDM-derived extracellular vesicles.**

The cells were cultured in exosome-free medium for 48 h, the culture medium was collected, and the extracellular particles were isolated. (A) The size (nm, nanometers) distribution of EVs was quantified via NTA. (B) Representative electron microscopy images; scale bar =100 nm. (C) Analysis of CD63 and CD81 expression in EVs by nanoflow cytometry. (D) Representative Western blots showing the expression of exosome markers and endoplasmic reticulum-specific proteins in total cell lysates and exosomes. The data are expressed as the mean ± SD of three independent experiments performed in triplicate and were analyzed by the t test (n=3).


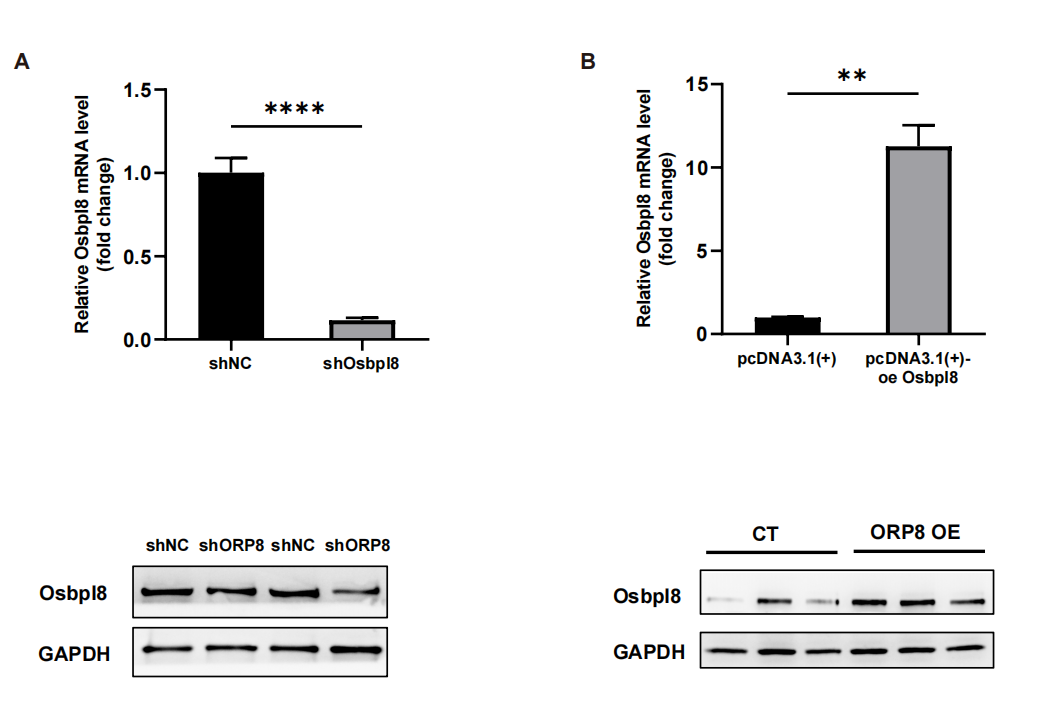


**Fig. S3. Transfection efficiency of Osbpl8 in primary murine hepatocytes.**

Cells were transfected with pcDNA3.1(+)/Ospbl8-pcDNA3.1(+)-C-Myc plasmid or Osbpl8-pLKO.1-copGFP-PURO shRNA/negative control (NC) shRNA for 36-48 hours. The Osbpl8 transfection efficiency was determined by (A) qRT‒PCR and (B) Western blotting. **, *p<*0.01; ****, *p<*0.0001

**
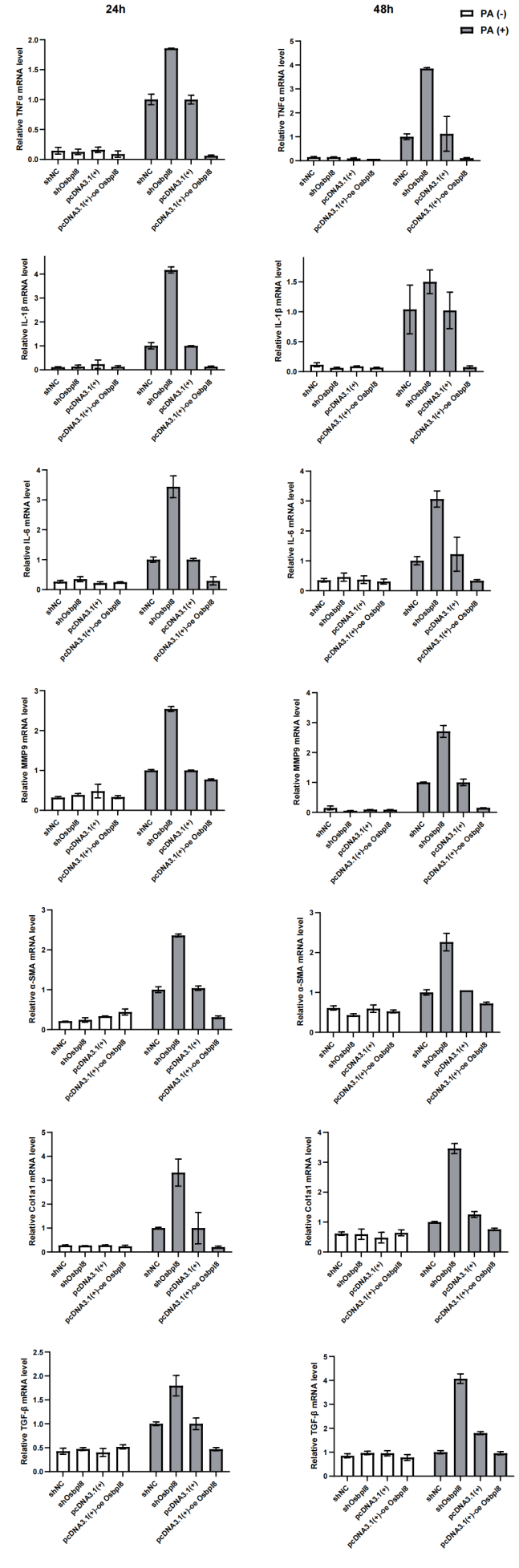
Fig. S4. Pro-inflammatory gene and pro-fibrotic gene mRNA expression detected by RT-qPCR supplementary for Fig. 4.** * *p<*0.05; **, *p<*0.01 ; ***, *p<*0.001; ****, *p<*0.0001 (n=3)

**Fig. S5. Intracellular reactive oxygen species (ROS) levels were detected by flow cytometry supplementary for Fig. 4 (Without PA treatment).**
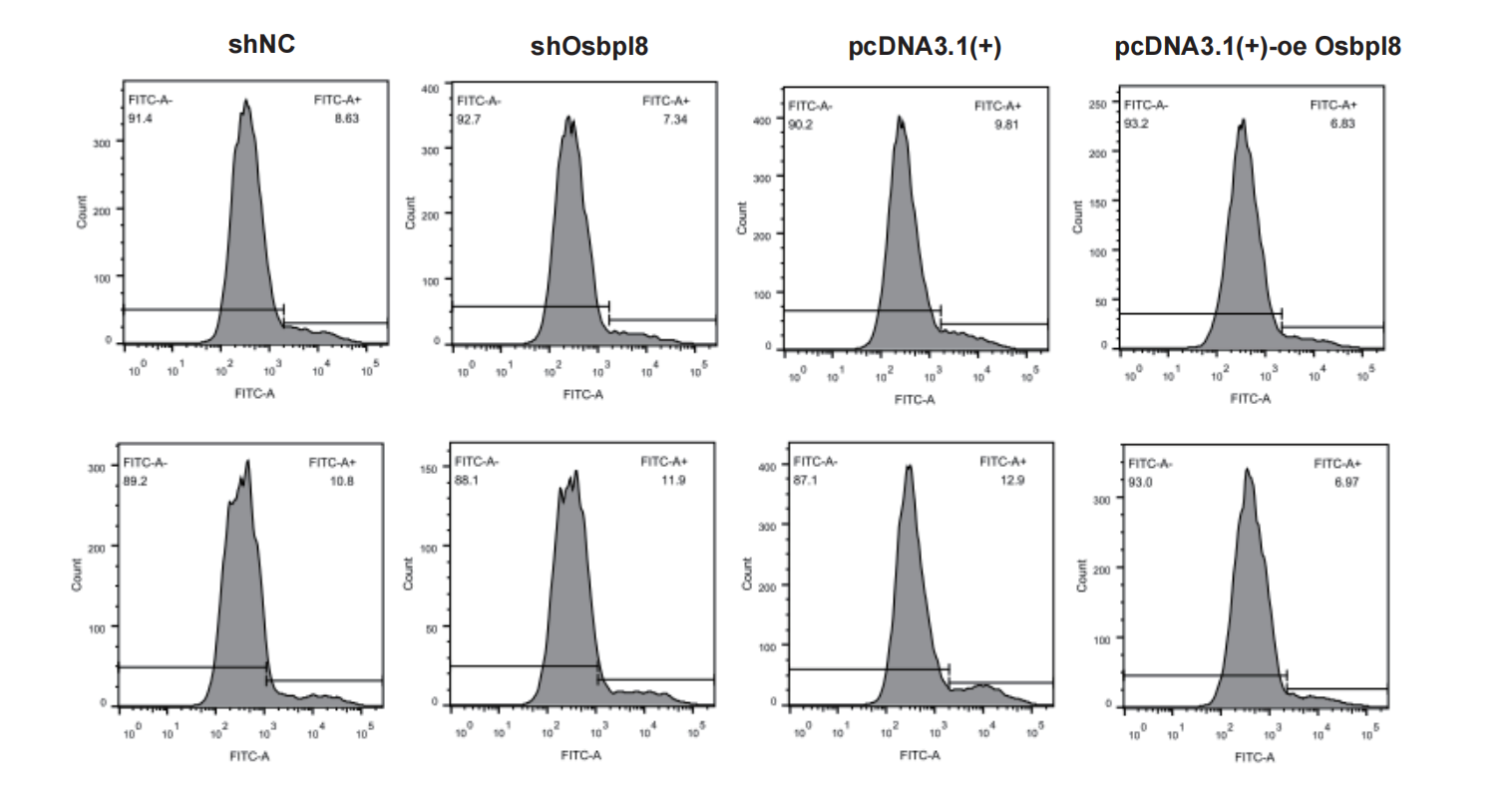


**
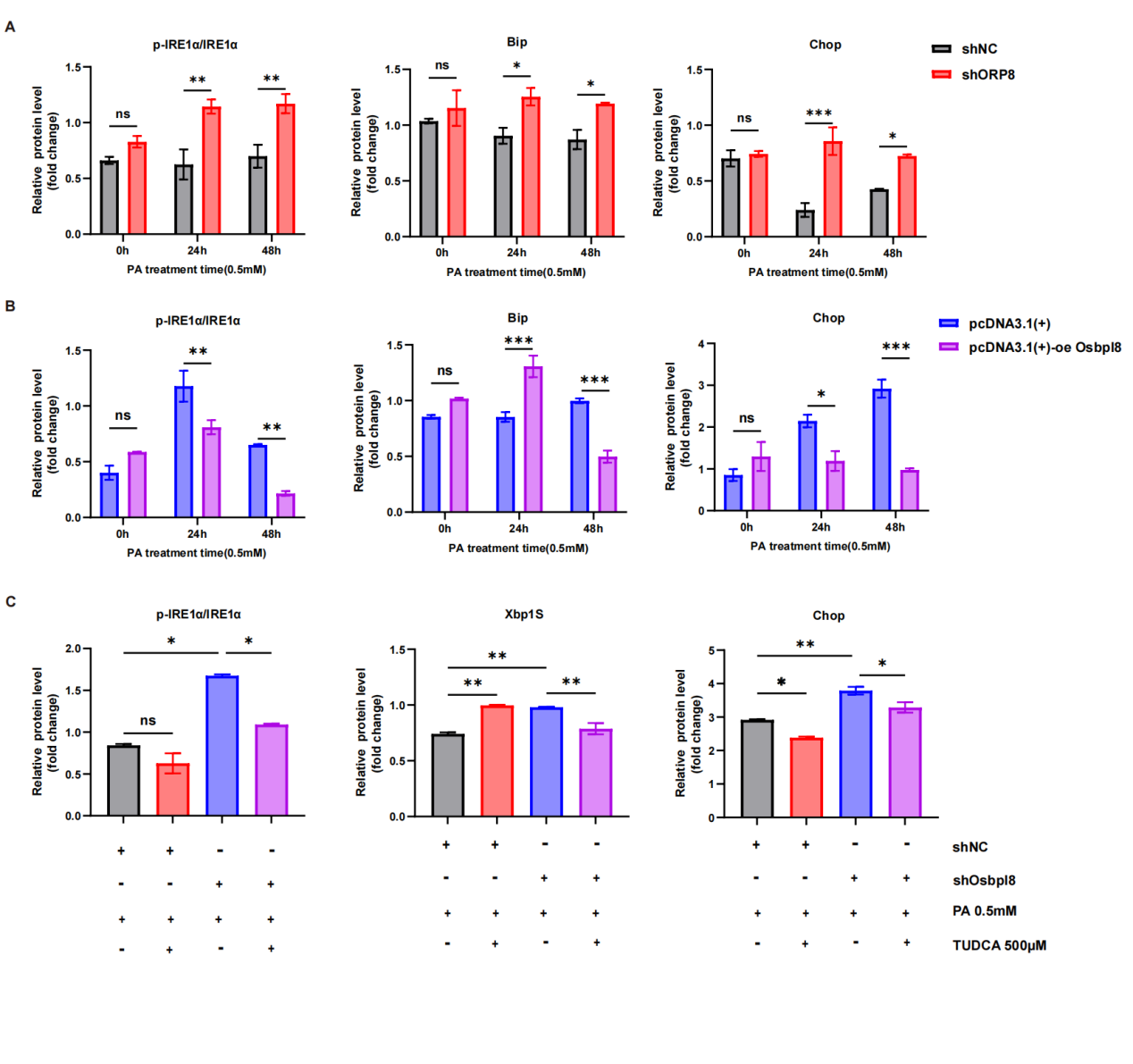
Fig. S6. The relative protein quantitative analysis for Fig. 5.** * *p<*0.05; **, *p<*0.01; ***, *p<*0.001

**
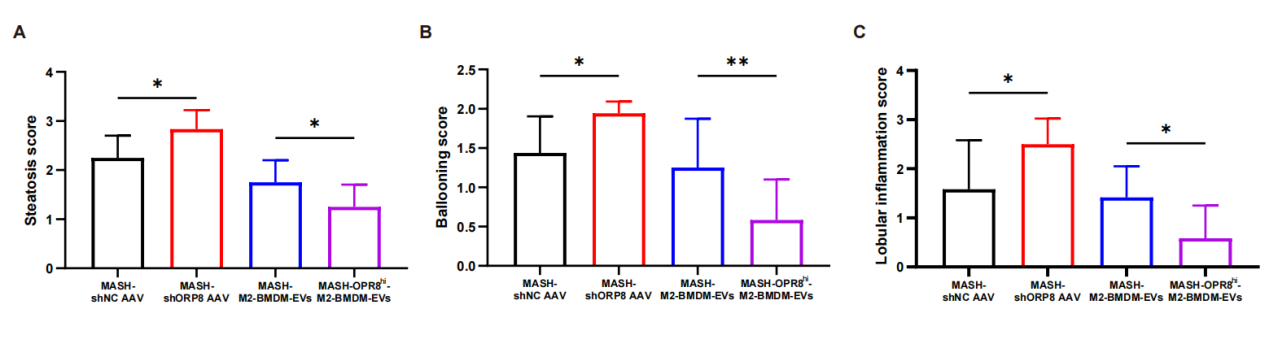
Fig. S7. Histopathological scores for (A) steatosis, (B) ballooning, (C) lobular inflammation for Fig.6B.** * *p<*0.05; **, *p<*0.01 (n=6-8)

**3. Supplementary table**

**Table S1 Primer List**

| **Name** | **Sequence 5’ 3’** | **Supplier** |
| --- | --- | --- |
| **18s rRNA-F** | GGACAGGACTAGGCGGAACA | Generay |
| **18s rRNA-R** | AGGGGAGAGCGGGTAAGAGA | Generay |
| **TNFα-F** | CCCTCACACTCAGATCATCTTCT | Generay |
| **TNFα-R** | CCCTCACACTCAGATCATCTTCT | Generay |
| **IL-1β-F** | GCAACTGTTCCTGAACTCAACT | Generay |
| **IL-1β-R** | ATCTTTTGGGGTCCGTCAACT | Generay |
| **IL-6-F** | TAGTCCTTCCTACCCCAATTTCC | Generay |
| **IL-6-R** | TTGGTCCTTAGCCACTCCTTC | Generay |
| **iNOS-F** | GTTCTCAGCCCAACAATACAAGA | Generay |
| **iNOS-R** | GTGGACGGGTCGATGTCAC | Generay |
| **CXCL2-F** | CCAACCACCAGGCTACAGG | Generay |
| **CXCL2-R** | GCGTCACACTCAAGCTCTG | Generay |
| **MMP9-F** | CTGGACAGCCAGACACTAAAG | Generay |
| **MMP9-R** | CTCGCGGCAAGTCTTCAGAG | Generay |
| **α-SMA-F** | GTCCCAGACATCAGGGAGTAA | Generay |
| **α-SMA-R** | TCGGATACTTCAGCGTCAGGA | Generay |
| **Col1a1-F** | GCTCCTCTTAGGGGCCACT | Generay |
| **Col1a1-R** | CCACGTCTCACCATTGGGG | Generay |
| **TGFβ-F** | CTCCCGTGGCTTCTAGTGC | Generay |
| **TGFβ-R** | GCCTTAGTTTGGACAGGATCTG | Generay |
| **Scd1-F** | TTCTTGCGATACACTCTGGTGC | Generay |
| **Scd1-R** | CGGGATTGAATGTTCTTGTCGT | Generay |
| **CD36-F** | ATGGGCTGTGATCGGAACTG | Generay |
| **CD36-R** | GTCTTCCCAATAAGCATGTCTCC | Generay |
| **PPARα-F** | AACATCGAGTGTCGAATATGTGG | Generay |
| **PPARα-R** | CCGAATAGTTCGCCGAAAGAA | Generay |
| **PPARγ-F** | GGAAGACCACTCGCATTCCTT | Generay |
| **PPARγ-R** | GTAATCAGCAACCATTGGGTCA | Generay |
| **Cpt1a-F** | CTCCGCCTGAGCCATGAAG | Generay |
| **Cpt1a-R** | CACCAGTGATGATGCCATTCT | Generay |
| **Fasn-F** | GGAGGTGGTGATAGCCGGTAT | Generay |
| **Fasn-R** | TGGGTAATCCATAGAGCCCAG | Generay |
| **Chop-F** | CTGGAAGCCTGGTATGAGGAT | Generay |
| **Chop-R** | CAGGGTCAAGAGTAGTGAAGGT | Generay |
| **Grp78-F** | ACTTGGGGACCACCTATTCCT | Generay |
| **Grp78-R** | ATCGCCAATCAGACGCTCC | Generay |
| **Xbp1-F** | AGCAGCAAGTGGTGGATTTG | Generay |
| **Xbp1-R** | GAGTTTTCTCCCGTAAAAGCTGA | Generay |
| **Ospbl8-F** | ATGGAGGCAGCCTTAGCAGA | Generay |
| **Ospbl8-R** | CAAATGCTGAGGTTCGTCACT | Generay |

**Table S2 Antibody and Reagent information**

| **Name** | **Supplier** | **Cat No.** | **Clone No.** |
| --- | --- | --- | --- |
| **TRIzol™** | Invitrogen™ | 15596026 |  |
| **HiScript III All-in-one RT SuperMix Perfect for qPCR** | Vazyme | R333-01 |  |
| **ChamQ Universal SYBR qPCR Master Mix** | Vazyme | Q711-02 |  |
| **ATP Determination Kit** | Thermo Fisher Scientific | A22066 |  |
| **TUNEL Assay Kit (Fluorescence, 488 nm)** | Cell Signaling Technology | 25879S |  |
| **Fluorometric Intracellular Ros Kit** | Sigma-Aldrich | MAK143-1KT |  |
| **LDH Cytotoxicity Assay Kit** | Cell Signaling Technology | 37291S |  |
| **Oil Red O** | Sigma-Aldrich | [O1391](https://www.sigmaaldrich.cn/CN/en/product/sigma/o1391) |  |
| **Nile Red** | Sigma-Aldrich | 19123 |  |
| **M-CSF/CSF1 Protein, Mouse, Recombinant** | TargetMol® | TMPY-00464 |  |
| **IL-4 Protein, Mouse, Recombinant** | TargetMol® | [TMPY-02558](https://www.targetmol.cn/recombinant-protein/il_4_protein_mouse_recombinant) |  |
| **IL-13 Protein, Mouse, Recombinant** | TargetMol® | [TMPY-03123](https://www.targetmol.cn/recombinant-protein/il_13_protein_mouse_recombinant) |  |
| **GAPDH (D16H11) XP® Rabbit mAb** | Cell Signaling Technology | 5174S |  |
| **β-Tubulin antibody** | Cell Signaling Technology | 2146S |  |
| **[Anti-ORP8 antibody](https://www.abcam.cn/products/primary-antibodies/orp8-antibody-ab228990.html)** | Abcam | ab228990 |  |
| **XBP1S-specific Polyclonal antibody** | Proteintech | 24868-1-AP |  |
| **Anti-IRE1 (phospho S724) antibody** | Abcam | ab124945 |  |
| **[Anti-IRE1 antibody](https://www.abcam.cn/products/primary-antibodies/ire1-antibody-ab37073.html)** | Abcam | ab37073 |  |
| **GRP78/BIP Polyclonal antibody** | Proteintech | 11587-1-AP |  |
| **CHOP Polyclonal antibody** | Proteintech | 15204-1-AP |  |
| **smooth muscle actin Polyclonal antibody** | Proteintech | 14395-1-AP |  |
| **Collagen Type I Monoclonal antibody** | Proteintech | 67288-1-Ig |  |
| **CD63 Polyclonal antibody** | Proteintech | 25682-1-AP |  |
| **CD81 Monoclonal antibody** | Proteintech | 66866-1-Ig |  |
| **TSG101 Polyclonal antibody** | Proteintech | 28283-1-AP |  |
| **ALT (SGPT) Liquid Reagents** | Pointe Scientific | 23-666-087 |  |
| **AST (SGOT) Liquid Reagents** | Pointe Scientific | 23-666-121 |  |
| **[Triglyceride Assay Kit](https://www.abcam.cn/products/assay-kits/triglyceride-assay-kit-quantification-ab65336.html)** | Abcam | ab65336 |  |
| **TUDCA** | TargetMol® | T2532 |  |
| **Palmitic acid** | Sigma-Aldrich | P0500 |  |
| **PE/Cyanine7 anti-mouse CD45 Antibody** | BioLegend | 103114 | [30-F11](https://www.biolegend.com/en-gb/search-results?Clone=30-F11) |
| **APC/Cyanine7 anti-mouse F4/80 Antibody** | BioLegend | 123118 | [BM8](https://www.biolegend.com/en-gb/search-results?Clone=BM8) |
| **Pacific Blue™ anti-mouse/human CD11b Antibody** | BioLegend | 01223 | [1/70](https://www.biolegend.com/en-gb/search-results?Clone=M1/70) |
| **Alexa Fluor® 647 anti-mouse Tim-4 Antibody** | BioLegend | 130007 | RMT4-54 |
| **FITC anti-mouse CD63 Antibody** | BioLegend | 143919 | [NVG-2](https://www.biolegend.com/en-gb/search-results?Clone=NVG-2) |
| **APC anti-mouse/rat CD81 Antibody** | BioLegend | 104909 | [Eat-2](https://www.biolegend.com/en-gb/search-results?Clone=Eat-2) |
| **LEGENDplex™ Mouse M1 Macrophage Panel (8-plex)** | BioLegend | 740847 |  |
